# Pattern mRNA expression similarities between ATL patients and patients coinfected with HTLV-I-Strongyolides stercoralis

**DOI:** 10.1186/1742-4690-8-S1-A165

**Published:** 2011-06-06

**Authors:** Michael Talledo, Giovanni López, Kristien Verdonck, Elsa González, Martin Tipismana, Eduardo Gotuzzo, Daniel Clark

**Affiliations:** 1 Inst de Med Trop Alexander von Humboldt, Univ. Peruana Cayetano Heredia, Lima, Peru; 2 Dept of Med Genetics, Univ. of Antwerp, Belgium; 3 Virology Unit, Dept. of Microbiol., Inst. of Tropical Med., Antwerp, Belgium; 4 Fac de Ciencias y Filosofía Lab de Inves y Des, Univ. Peruana Cayetano Heredia, Lima, Peru

## Introduction

HTLV-I is associated to Adult T-cell leukemia (ATL) characterized by genetic disorders and an altered mRNA expression. Individuals who have a coinfection between HTLV-I and Strongyolides stercoralis (SS) might have an increased risk to develop ATL. The mechanism for the synergism between these two infectious agents is unclear. In this study we evaluate the mRNA expression in Peripheral Blood Mononuclear Cells (PBMC) from three different groups, to determine mRNA profiles similarities or divergences among patients with a)ATL, b)HTLV-I-SS and c)Asymptomatic carriers (AC).

## Subjects methods

mRNA expression of eighty genes involved in the DNA damage pathway (Sabiosciences-Qiagen) were evaluated among HTLV-1 positive patients: a)ATL (n=6), b)HTLV-I-SS (n=8) and c)Asymptomatic carriers(n=6). RNA was extracted from PBMCs and analyzed by using PCR superarray plates from Sabiosciences-Qiagen, one plate was used to analyze 80 genes for every patient. Sabiosciences sample sheet was used for calculations.

## Results

The number of genes differentially expressed among these groups was: a) nine genes between ATL and AC, P values range 0.045 to 0.0073, b) nine genes between HTLV-I—SS and AC, P values range 0.0497 to 0.0098 and c) two genes between ATL and SS, P values 0.027 and 0.046.

## Conclusion

Two genes, the G-2 and S-phase expressed 1 gene and the Fanconi anemia, complementation group G gene were commonly increased in ATL and HTLV-I—SS compared to AC. Similar profiles of mRNA expression regarding of DNA damage pathway were observed among ATL and HTLV-I—SS groups, suggesting some shared regulatory mechanisms during infection in vivo.

